# Order effects in high stakes undergraduate examinations: an analysis of 5 years of administrative data in one UK medical school

**DOI:** 10.1136/bmjopen-2016-012541

**Published:** 2016-10-11

**Authors:** Jenni Burt, Gary Abel, Matt Barclay, Robert Evans, John Benson, Mark Gurnell

**Affiliations:** 1 Primary Care Unit, Cambridge Centre for Health Services Research, Cambridge, UK; 2 University of Exeter Medical School, Exeter, UK; 3 Primary Care Unit, Institute of Public Health, Cambridge, UK; 4 Wellcome Trust-MRC Institute of Metabolic Science, NIHR Cambridge Biomedical Research Centre & University of Cambridge School of Clinical Medicine, Cambridge, UK

**Keywords:** MEDICAL EDUCATION & TRAINING, Educational Measurement, Clinical Competence

## Abstract

**Objective:**

To investigate the association between student performance in undergraduate objective structured clinical examinations (OSCEs) and the examination schedule to which they were assigned to undertake these examinations.

**Design:**

Analysis of routinely collected data.

**Setting:**

One UK medical school.

**Participants:**

2331 OSCEs of 3 different types (obstetrics OSCE, paediatrics OSCE and simulated clinical encounter examination OSCE) between 2009 and 2013. Students were not quarantined between examinations.

**Outcomes:**

(1) Pass rates by day examination started, (2) pass rates by day station undertaken and (3) mean scores by day examination started.

**Results:**

We found no evidence that pass rates differed according to the day on which the examination was started by a candidate in any of the examinations considered (p>0.1 for all). There was evidence (p=0.013) that students were more likely to pass individual stations on the second day of the paediatrics OSCE (OR 1.27, 95% CI 1.05 to 1.54). In the cases of the simulated clinical encounter examination and the obstetrics and gynaecology OSCEs, there was no (p=0.42) or very weak evidence (p=0.099), respectively, of any such variation in the probability of passing individual stations according to the day they were attempted. There was no evidence that mean scores varied by day apart from the paediatric OSCE, where slightly higher scores were achieved on the second day of the examination.

**Conclusions:**

There is little evidence that different examination schedules have a consistent effect on pass rates or mean scores: students starting the examinations later were not consistently more or less likely to pass or score more highly than those starting earlier. The practice of quarantining students to prevent communication with (and subsequent unfair advantage for) subsequent examination cohorts is unlikely to be required.

**Strengths and limitations of this study**

- The study data set, containing 5 years of data from three separate objective structured clinical examinations (OSCE) in one medical school, is the largest to date to be analysed to investigate the impact of examination schedule on examination performance.
- The varying nature of examinations between medical schools makes it challenging to conduct cross-institutional analyses, but the inclusion of only one institution may limit the generalisability of our findings.
- Ideally, we would consider the impact of examiners on variations in examination performance according to day: however, this is not straightforward to satisfactorily accomplish as examiner effects may be confounded by subject and station difficulty, and could change across different years.

## Introduction

High stakes undergraduate medical assessments determine whether a student may or may not progress to medical qualification. As such, it is essential that the examination processes are valid, reliable, transparent, and fair. Medical schools worldwide use objective structured clinical examinations (OSCEs)^1^ ^2^ in this context to assess students’ clinical and communication skills. Such examinations aspire to ensure robust procedures by requiring all candidates to undertake the same clinical stations, to be completed within predetermined time limits and assessed using the same marking scheme.^2^ To accommodate all candidates for these examinations, many medical schools are compelled to run an OSCE repeatedly, with students scheduled to undertake the examination in sequential groups over a number of days. This leads to two concerns for students and medical schools.

The first concern relates to security breaches: it is possible that students in earlier scheduled examination times might tell those in later times about the content of the examination, either advantaging (or potentially disadvantaging) those students who come later. To reduce the potential for this, some medical schools routinely quarantine earlier student examination groupings from later ones until an overall OSCE is complete. This involves challenges for the medical schools in accommodating quarantined students (especially if an OSCE runs over more than 1 day) and in ensuring effective quarantine, such as restricting the use of smart phones/watches and other mobile devices. In addition, it imposes a significant burden on quarantined students, which is not shared by their colleagues in later cohorts.

With OSCEs’ focus on assessment of skills, rather than knowledge, some have argued that the short time lag between examination groupings is insufficient for any briefing about examination content to lead to an improvement in the performance of candidates in later examination times.^3^ ^4^ For example, a 1989 study reported no evidence of information sharing affecting the performance among US fourth year medical students in OSCEs (which, in this instance, took place over a period of several weeks).^5^ Others remain concerned about the likely impact of security breaches.^6^ Collusion between third year medical students on the content of OSCEs has previously been identified through monitoring of discussions between students on an electronic discussion board, in which concerns were expressed about this taking place.^7^ An experimental study modelling the effect of a severe security breach (ie, the leaking of checklists or the provision of coaching for three of the six clinical stations) found that students who had received additional information outperformed the control group by around 7%.^8^

The second concern relates to whether examination grouping, in itself and independent of possible security breaches, may influence a candidate's performance. One explanation for this is that students in different examination groups may perform variably as a result of psychological pressures relating to the timing of their examination or changes in aspects of examination process between groups. An additional or alternative explanation is that examiners’ scoring may change over time, becoming either more or less generous across examination groupings. The core question here is whether there is something inherent about being in the first versus subsequent examination groups that places candidates at an advantage or disadvantage in comparison to their peers.

Small but inconsistent effects have previously been found for the time of day (morning or afternoon) and day on which second-year medical students undertake OSCEs in a US medical school, assessed on a pass/fail basis.^9^ However, other research has demonstrated little impact of the day of the examination (in dental OSCEs in the Netherlands,^10^ in undergraduate medical OSCEs in Spain^11^) or the timing of the examination (in undergraduate medical OSCEs in Canada^12^). These studies were relatively small in scale (with 772,^9^ 463,^10^ 172^11^ and 69^12^ students, respectively) and examined a variety of OSCE set-ups, including the use of parallel streams^12^ and non-consecutive days with no quarantining of students.^10^

Given continuing concern about this issue, there is a need for larger-scale and up-to-date examination of the impact of examination order on performance in modern OSCE settings. The purpose of this study was, therefore, to investigate the association between the scores achieved by students in high-stakes OSCEs in one UK medical school, the University of Cambridge School of Clinical Medicine, and the examination grouping to which they were assigned.

## Methods

We conducted a retrospective analysis of three high-stakes OSCEs conducted in the School of Clinical Medicine between 2009 and 2013. Students in sequential examination groupings were not quarantined in any of these examinations. Data were retrieved by a single member of the research team, with the assistance of the School examinations administration team. Student and examiner identities were removed on data retrieval and replaced by a unique, anonymised study identifier. Our data covered:

1. Year 5 obstetrics and gynaecology OSCE: for this examination, individual candidates sat all stations in 1 day and in each year the OSCE was completed over 2 consecutive days.
2. Year 5 paediatrics OSCE: for this examination, individual candidates sat all stations in 1 day and in each year the OSCE was completed over 2 consecutive days.
3. Year 6 simulated clinical encounter examination (SCEE) OSCE: for this examination, individual candidates sat stations over 2 days and in each year the OSCE was completed in 3 days. Candidates were evenly split between sitting stations on days 1 and 2, days 1 and 3, and days 2 and 3, with the same number of candidates taking each station on each day.

For all OSCEs, the content of each station, including the question wording, did not vary between circuits or between days.

### Data

For each OSCE examination, data were obtained for individual candidates and individual stations, alongside the overall pass mark for each examination in each year. Data were obtained for each OSCE for 2009–2013. For each candidate, we knew whether they passed the examination, their mean score, whether they passed each individual station of the examination and their score for each individual station. We had further information on the subject of each station, a pseudonymous code for the examiner for each station and the day and time of each station. There were no missing data.

In the case of all three examinations, in order to pass, students were required (1) to meet the overall examination pass mark, as defined by the borderline group method,^13^ and (2) to additionally pass a minimum of 50% of individual stations: this ensures that poor performance in several stations cannot be compensated for by exceptionally high performance in one or two other stations.

### Statistical analysis

We used a series of models to investigate various aspects of potential order effects in OSCEs, which we applied separately to each of the three examinations under consideration ((1) Year 5 obstetrics and gynaecology OSCE, (2) Year 5 paediatrics OSCE and (3) Year 6 simulated clinical encounter examination OSCE). Each model included data from all candidates and all years for that OSCE.

Our primary question was whether the probability of passing each OSCE varied according to the day on which the examination was undertaken. We used a logistic regression model, adjusting for the year of examination, to investigate whether the probability of passing each OSCE varied according to the day on which each candidate started the examination (Model 1). We recognise, however, that the very high overall pass rate (>97%) limits the power of this approach. For example, if the true pass rate on day 1 was 96.5%, and the true pass rate on day 2 was 98.5%, the power to detect this difference, an OR of 2.4, would be <50%.

Our second question (less limited by power than our primary question) was whether the probability of passing individual stations varied according to the day on which those stations were attempted. This differs from Model 1, which considered the first day on which candidates attempted any stations (remembering that, in some OSCEs, candidates were required to be examined over 2 days). We used a mixed-effects logistic regression model adjusting for year (fixed effect) and for clustering of individual station results within candidates using a random effect (Model 2).

Our third question was whether any observed effects of the day on which stations were attempted were consistent from year to year: to consider this, we augmented Model 2 by including an interaction between day and the year-cohort (Model 3).

The above analyses focus on the nature of these OSCEs as pass or fail exams; as long as candidates do well enough to pass, their actual overall score is not of great importance. However, in the University of Cambridge Medical School, as elsewhere, candidates may be awarded a Pass with Distinction if they score particularly highly, with the potential for subsequent impact on their career opportunities. We, therefore, used a linear regression model to investigate whether there were differences in the overall mean score between start days of the OSCE (as in Model 1), adjusting for year (Model 4).

## Results

Between 2009 and 2013, 770 candidates sat a Year 5 obstetrics and gynaecology OSCE, 771 candidates sat a Year 5 paediatrics OSCE, and 790 candidates sat a Year 6 SCEE OSCE at Cambridge. Around 150–160 candidates sat each examination in each year.

Overall pass rates were very high: of 2331 total examinations taken across all three OSCEs, 2273 (97%) were passed (table 1). Pass rates appeared broadly similar across start days, as did mean scores. Pass rates for individual stations were lower, with passes given for 17 421 (87%) of the 20 078 stations taken.

**Table 1:** Descriptive statistics for the SCEE, stage 2 obstetrics and gynaecology and stage 2 paediatrics OSCE results, 2009–2013

	Start day 1		Start day 2				Total	
Candidates	Failed (%)	Passed (%)	Failed (%)	Passed (%)			Failed (%)	Passed (%)
SCEE	14 (2.7)	514 (97.3)	4 (1.5)	258 (98.5)			18 (2.3)	772 (97.7)
Obs and Gynae	14 (3.7)	369 (96.3)	17 (4.4)	370 (95.6)			31 (4.0)	739 (96.0)
Paeds	7 (1.8)	383 (98.2)	2 (0.5)	379 (99.5)			9 (1.2)	762 (98.8)
	**Start day 1**		**Start day 2**				**Total**	
**Mean score (%)**	**Mean (SD)**		**Mean (SD)**				**Mean (SD)**	
SCEE	70.8 (7.8)		70.7 (7.7)				70.7 (7.7)	
Obs and Gynae	69.5 (7.9)		69.3 (8.8)				69.4 (8.3)	
Paeds	68.1 (8.3)		70.0 (8.0)				69.0 (8.2)	
	**Day 1**		**Day 2**		**Day 3**		**Total**	
**Stations**	**Failed (%)**	**Passed (%)**	**Failed (%)**	**Passed (%)**	**Failed (%)**	**Passed (%)**	**Failed (%)**	**Passed (%)**
SCEE	363 (13.8)	2277 (86.3)	353 (13.4)	2277 (86.6)	323 (12.3)	2307 (87.7)	1039 (13.1)	6867 (86.9)
Obs and Gynae	404 (14.2)	2433 (85.8)	464 (16.2)	2401 (83.8)			868 (15.2)	4834 (84.8)
Paeds	418 (12.8)	2852 (87.2)	332 (10.4)	2868 (89.6)			750 (11.6)	5720 (88.4)

Gynae, gynaecology; Obs, obstetrics; Paeds, paediatrics; SCEE, simulated clinical encounter examination.

### Probability of passing each examination according to start day (Model 1)

We found no evidence that pass rates differed according to the day on which the examination was started by a candidate (p>0.1, table 2). However, as noted above, the high pass rates limit the power of this analysis to detect any notable difference between days (reflected in the wide CIs, particularly for the SCEE and paediatrics OSCEs).

**Table 2:** Probability of passing the OSCE for the SCEE, stage 2 obstetrics and gynaecology and stage 2 paediatrics OSCE results, 2009–2013 (model 1)

		SCEE		Obs and Gynae		Paeds	
	Factor	OR (95% CI)	p Value	OR (95% CI)	p Value	OR (95% CI)	p Value
First day	Day 1	Reference	0.327	Reference	0.575	Reference	0.121
	Day 2	1.76 (0.57 to 5.42)		0.81 (0.39 to 1.68)		3.49 (0.72 to 16.94)	
Year	2009	*	0.254	Reference	0.002	Reference	0.121
	2010	1.41 (0.23 to 8.57)		1.52 (0.62 to 3.72)		1.12 (0.15 to 8.06)	
	2011	0.37 (0.09 to 1.40)		4.77 (1.32 to 17.26)		2.26 (0.20 to 25.26)	
	2012	0.61 (0.14 to 2.61)		2.76 (0.95 to 8.05)		2.19 (0.20 to 24.45)	
	2013	Reference		7.13 (1.57 to 32.42)		0.73 (0.12 to 4.44)	

*All candidates in 2009 passed the SCEE OSCE, so this year was omitted from the model. Gynae, gynaecology; Obs, obstetrics; OSCE, objective structured clinical examinations; Paeds, paediatrics; SCEE, simulated clinical encounter examination.

### Probability of passing individual stations according to the day on which those stations were attempted (Model 2)

We found no evidence of a difference in the probability of passing an individual station in the SCEE OSCE according to the day on which that station was attempted (p=0.42, table 3). For the obstetrics and gynaecology OSCE, we found very weak evidence of a difference according to the day on which each station was attempted (p=0.1, table 3), with a lower probability of passing an individual station on the second day (OR 0.85, 95% CI 0.70 to 1.03). Finally, in the paediatrics OSCE, we found evidence of a difference according to the day on which each station was attempted (p=0.01, table 2), with a higher probability of passing an individual station on the second day (OR 1.27, 95% CI 1.05 to 1.54).

**Table 3:** Probability of passing individual stations for the SCEE, stage 2 obstetrics and gynaecology and stage 2 paediatrics OSCE results, 2009–2013 (model 2)

	SCEE		Obs and Gynae		Paeds	
Factor	OR (95% CI)	p Value	OR (95% CI)	p Value	OR (95% CI)	p Value
Day of station						
Day 1	Reference	0.424	Reference	0.099	Reference	0.013
Day 2	1.09 (0.92 to 1.30)		0.85 (0.70 to 1.03)		1.27 (1.05 to 1.54)	
Day 3	1.12 (0.94 to 1.34)					
Year						
2009	Reference	<0.001	Reference	0.038	Reference	0.524
2010	0.78 (0.58 to 1.04)		1.19 (0.88 to 1.62)		0.85 (0.63 to 1.16)	
2011	0.63 (0.48 to 0.83)		1.31 (0.97 to 1.78)		1.01 (0.74 to 1.38)	
2012	0.98 (0.84 to 1.51)		1.48 (1.09 to 2.01)		0.90 (0.67 to 1.23)	
2013	1.13 (0.84 to 1.51)		1.55 (1.14 to 2.10)		0.81 (0.61 to 1.09)	
Candidate						
SD of random effect	0.80 (0.70 to 0.92)		0.80 (0.68 to 0.93)		0.71 (0.59 to 0.86)	
95% reference range†	23.18 (15.54 to 36.66)		22.76 (14.36 to 39.06)		16.48 (10.22 to 29.33)	

†Odds ratio comparing the probability of passing for a candidate better than 97.5% of other candidates to that of a candidate worse than 97.5% of other candidates, estimated using standard deviation of the random effect (95% reference range = *e*^*2×1.96×SDRE*^). Gynae, gynaecology; Obs, obstetrics; Paeds, paediatrics; SCEE, simulated clinical encounter examination.

The size of these differences can be contextualised by comparing them to the year-to year differences and to the variability between candidates. The differences between the day on which the stations were attempted were smaller than the differences between year-cohorts (table 3), and much smaller than the variability between candidates (captured by the random effect). Using the SD of the random effect we estimate ORs comparing the ‘best’ candidates (defined as those who are better at passing OSCE stations than 97.5% of other candidates) to the ‘worst’ candidates (defined as those worse at passing OSCE stations than 97.5% of other candidates at between 16.48 (paediatrics OSCE) and 23.18 (SCEE OSCE).

### Probability of passing individual stations according to the day on which those stations were attempted, within each examination year (Model 3)

All three OSCEs had at least 1 year in which we found a large, highly statistically significant difference in the odds of passing individual stations according to the day on which stations were attempted (table 4). For the SCEE and the obstetrics and gynaecology OSCEs, the higher probability of passing on a particular day in 1 year were matched by a lower probability of passing on that day in a different year. For example, in the 2010 SCEE, candidates’ odds of passing stations on day 2 were twice those on day 1, but in the 2012 SCEE candidates’ odds of passing stations on day 2 were half those on day 1. For the paediatrics OSCE there was a significant increase in pass rate for day two (p<0.001) in 2010 only: for all other years there was no clear evidence of a difference in pass rates between days.

**Table 4:** Probability of passing individual stations by day within individual years for the SCEE, stage 2 obstetrics and gynaecology and stage 2 paediatrics OSCE results, 2009–2013 (model 3)

	SCEE*		Obs and Gynae*		Paeds**	
Factor	OR (95% CI)	p Value	OR (95% CI)	p Value	OR (95% CI)	p Value
2009 Day of station						
Day 1	Reference	0.717	Reference	0.559	Reference	0.626
Day 2	1.05 (0.70 to 1.56)		0.88 (0.58 to 1.35)		1.11 (0.72 to 1.72)	
Day 3	1.18 (0.78 to 1.79)					
2010 Day of station						
Day 1	Reference	<0.001	Reference	0.539	Reference	<0.001
Day 2	2.08 (1.38 to 3.15)		1.14 (0.75 to 1.73)		2.15 (1.39 to 3.31)	
Day 3	1.09 (0.75 to 1.59)					
2011 Day of station						
Day 1	Reference	0.736	Reference	0.011	Reference	0.274
Day 2	1.05 (0.74 to 1.50)		1.74 (1.14 to 2.67)		1.28 (0.82, 1.98)	
Day 3	1.15 (0.68 to 1.62)					
2012 Day of station						
Day 1	Reference	<0.001	Reference	<0.001	Reference	0.988
Day 2	0.53 (0.36 to 0.78)		0.48 (0.31 to 0.74)		1.00 (0.65 to 1.53)	
Day 3	1.05 (0.68 to 1.62)					
2013 Day of station						
Day 1	Reference	0.243	Reference	0.003	Reference	0.615
Day 2	1.45 (0.94 to 2.23)		0.52 (0.34 to 0.80)		1.11 (0.74 to 1.65)	
Day 3	1.19 (0.78 to 1.80)					
Candidate						
SD of random effect	0.81 (0.70 to 0.93)		0.76 (0.64 to 0.90)		0.71 (0.58 to 0.85)	
95% reference range†	23.71 (15.84 to 37.65)		19.75 (12.46 to 34.07)		15.86 (9.82 to 28.32)	

*Combined p-value for day-year interaction: p<0.001. **Combined p-value for day-year interaction: p=0.103. †Odds ratio comparing the probability of passing for a candidate better than 97.5% of other candidates to that of a candidate worse than 97.5% of other candidates, estimated using the standard deviation of the random effect (95% reference range = *e*^*2×1.96×SDRE*^). Gynae, gynaecology; Obs, obstetrics; Paeds, paediatrics; SCEE, simulated clinical encounter examination.

### Differences in overall mean scores between start days of the OSCE, adjusting for year (model 4)

Candidates who sat the paediatrics OSCE on the second day of the examination tended to have higher scores, with a mean score difference of 1.8 (95% CI 0.8 to 2.8) (table 5). We note, however, that this is small compared to the SD of paediatrics OSCE scores (8.2).

**Table 5:** Mean difference in overall score by day and year for the SCEE, stage 2 obstetrics and gynaecology and stage 2 paediatrics OSCE results, 2009–2013 (model 4)

	SCEE		Obs and Gynae		Paeds	
Factor	Difference (95% CI)	p Value	Difference (95% CI)	p Value	Difference (95% CI)	p Value
First day						
Day 1	Reference	0.858	Reference	0.716	Reference	<0.001
Day 2	−0.09 (−1.13 to 0.94)		−0.21 (−1.33 to 0.91)		1.78 (0.79 to 2.76)	
Year						
2009	Reference	<0.001	Reference	<0.001	Reference	<0.001
2010	1.64 (0.008 to 3.20)		2.47 (0.67 to 4.27)		−2.36 (−3.94 to −0.78)	
2011	−1.56 (−3.09 to −0.03)		−0.30 (−2.10 to 1.49)		−10.35 (−11.93 to −8.77)	
2012	5.44 (3.92 to 6.96)		−0.05 (−1.85 to 1.76)		−9.30 (−10.89 to −7.71)	
2013	−4.56 (−6.09 to −3.02)		−5.10 (−6.90 to −3.30)		−9.09 (−10.67 to −7.51)	

Gynae, gynaecology; Obs, obstetrics; Paeds, paediatrics; SCEE, simulated clinical encounter examination.

There was no evidence that mean scores on either the SCEE or obstetrics and gynaecology OSCE varied between candidates starting on the first and second days of the examination (table 5).

## Discussion

While our analyses identified some potential order effects in the OSCEs under investigation, these were inconsistent in direction across the three examinations and relatively small. For all OSCEs, there was no evidence that overall pass rates varied according to the day on which the examination was started, although our certainty is limited by the very high overall pass rates. We did not seek to see if overall order of examinations taken made a difference; however, if this had been the case, we would expect it to have been highlighted in our analysis by the day the examination was started.

There was some evidence for variations in pass rates for individual stations according to the day on which stations were attempted. In particular, we found that in a number of years, there were strong differences in the chance of passing individual stations according to when they were taken. However, these effects were highly inconsistent across years and tended to cancel each other out when considering the 5 years of data together. Only the paediatric OSCE provided any substantive evidence of an order effect over the 5-year period, and even then it was dominated by a single year. The inconsistencies between examinations and years suggest that differences were unlikely to represent a true order effect, but rather other factors that varied between years. These could potentially be attributable to different examiners, but could also be attributable to uncontrollable factors such as the weather or the traffic (see below). It is also noteworthy that the differences by day were smaller than the year-to-year differences and much smaller than the differences between candidates.

A particular strength of this analysis is the inclusion of large numbers of candidates and stations, across 5 years of the conduct of three separate examinations with no differences in content between days for each examination within each year. The completeness and level of detail of data available on candidates, stations, and examinations enabled us to undertake a comprehensive study of the association between the time of examination and overall pass rates and mean scores. The varying nature of examinations between medical schools makes it challenging to conduct cross-institutional analyses, although the inclusion of only one institution may limit the generalisability of our findings. We acknowledge that differences between examiners may explain some of the observed differences between days, and ideally we would consider the impact of potential confounding by examiner. However, this is not straightforward to satisfactorily accomplish: examiner effects may be confounded by subject and station difficulty, and could change across different years. Additionally, the simulated patients and patients used within the examinations may vary between circuits and days; we were not able to investigate the potential impact of this, as we did not hold information about the simulated patients and patients involved in these examinations. However, we note that all simulated patients are trained to a high standard and discuss each station in detail in advance to minimise variation in performance.

Our findings reflect those of the next largest analyses of OSCEs conducted on this issue to date, in which the effect of day and time of examination was examined over 4 years of second-year medical pass/fail examinations (1990–1993) in one US medical school.^9^ Here, while differences in the pass rate were identified according to the day of examination and the time of examination (morning or afternoon), none of these were consistent between years.^9^ The authors concluded that there was little evidence that test security between repeated examinations was a concern.^9^ Two other studies examining the effect of day on OSCE pass rates found no strong evidence of variation: one, an examination of dental OSCEs in 463 students across 4 days of the week,^10^ the second involving 172 final year medical students from three medical schools across 8 days.^11^ We can only speculate for the causes of inconsistent within-year, by-day variations in examination performance in our analyses. However, we would suggest that issues such as variations in the weather according to examination days are not entirely far-fetched as potential explanations. For example, psychological experiments have demonstrated that weather may affect a wide range of moods and behaviours, including risk-aversion,^14^ memory^15^ and concentration,^16^ all of which might affect  the performance of either students or examiners. The variations in the odds of passing between years are more reasonably likely to be as a result of differences in cohort ability.

These findings demonstrate clearly that there is little need to quarantine students across different cohorts of OSCE examinations. Such quarantining is recommended in some quarters,^17^ yet it is costly in time and money. It is apparent that, even though students in later cohorts may have the opportunity to discuss and review examination content with those who have already undertaken the examination, such discussions—if they occur—do not significantly affect the performance in examinations.
